# Identifying Objective Physiological Markers and Modifiable Behaviors for Self-Reported Stress and Mental Health Status Using Wearable Sensors and Mobile Phones: Observational Study

**DOI:** 10.2196/jmir.9410

**Published:** 2018-06-08

**Authors:** Akane Sano, Sara Taylor, Andrew W McHill, Andrew JK Phillips, Laura K Barger, Elizabeth Klerman, Rosalind Picard

**Affiliations:** ^1^ Affective Computing Group Media Lab Massachusetts Institute of Technology Cambridge, MA United States; ^2^ Brigham and Women’s Hospital Boston, MA United States; ^3^ Harvard Medical School Boston, MA United States

**Keywords:** mobile health, mood, machine learning, wearable electronic devices, smartphone, mobile phone, mental health, psychological stress

## Abstract

**Background:**

Wearable and mobile devices that capture multimodal data have the potential to identify risk factors for high stress and poor mental health and to provide information to improve health and well-being.

**Objective:**

We developed new tools that provide objective physiological and behavioral measures using wearable sensors and mobile phones, together with methods that improve their data integrity. The aim of this study was to examine, using machine learning, how accurately these measures could identify conditions of self-reported high stress and poor mental health and which of the underlying modalities and measures were most accurate in identifying those conditions.

**Methods:**

We designed and conducted the 1-month SNAPSHOT study that investigated how daily behaviors and social networks influence self-reported stress, mood, and other health or well-being-related factors. We collected over 145,000 hours of data from 201 college students (age: 18-25 years, male:female=1.8:1) at one university, all recruited within self-identified social groups. Each student filled out standardized pre- and postquestionnaires on stress and mental health; during the month, each student completed twice-daily electronic diaries (e-diaries), wore two wrist-based sensors that recorded continuous physical activity and autonomic physiology, and installed an app on their mobile phone that recorded phone usage and geolocation patterns. We developed tools to make data collection more efficient, including data-check systems for sensor and mobile phone data and an e-diary administrative module for study investigators to locate possible errors in the e-diaries and communicate with participants to correct their entries promptly, which reduced the time taken to clean e-diary data by 69%. We constructed features and applied machine learning to the multimodal data to identify factors associated with self-reported poststudy stress and mental health, including behaviors that can be possibly modified by the individual to improve these measures.

**Results:**

We identified the physiological sensor, phone, mobility, and modifiable behavior features that were best predictors for stress and mental health classification. In general, wearable sensor features showed better classification performance than mobile phone or modifiable behavior features. Wearable sensor features, including skin conductance and temperature, reached 78.3% (148/189) accuracy for classifying students into high or low stress groups and 87% (41/47) accuracy for classifying high or low mental health groups. Modifiable behavior features, including number of naps, studying duration, calls, mobility patterns, and phone-screen-on time, reached 73.5% (139/189) accuracy for stress classification and 79% (37/47) accuracy for mental health classification.

**Conclusions:**

New semiautomated tools improved the efficiency of long-term ambulatory data collection from wearable and mobile devices. Applying machine learning to the resulting data revealed a set of both objective features and modifiable behavioral features that could classify self-reported high or low stress and mental health groups in a college student population better than previous studies and showed new insights into digital phenotyping.

## Introduction

### Background

Recent advances in wearable and mobile technologies have enabled individuals to monitor their daily lives and enabled scientific investigators to passively collect real-time data without disrupting people’s habitual routines. Two examples of such devices are wrist-wearable devices that collect activity and other physiological data (eg, activity or sleep; heart rate; skin conductance, SC; blood pressure; and blood sugar level) and mobile phones (eg, smartphones) that monitor location, activity, social interaction over calls and texts (short message service, SMS), app use, screen on or off, and environmental data such as ambient light exposure and humidity.

Leveraging data from wearable and mobile devices to gain meaningful information about human health has been called digital phenotyping [1-3]. Digital phenotyping is defined as the moment-by-moment quantification of the individual-level human phenotype *in situ* using data from personal digital devices. Data from personal digital devices may be used to understand health and behaviors with a goal of preventing or minimizing disorders and diseases. For example, current health status, behavior history, and potential future health trajectories information might help (1) individuals become more aware of their risk profiles and enable them to make better informed decisions and take actions to change their behaviors to reduce potential negative physical and mental outcomes and (2) clinicians monitor changes in their client’s or patient’s status.

Mobile phones have been used to monitor stress and mental health [4-10]. The pioneering Student Life study that monitored 48 college students across a 10-week term using objective Android mobile phone sensors and usage investigated the relationship between well-being measures such as self-reported stress, depression, flourishing and loneliness, and academic performance [4]. Lower Perceived Stress Scale (PSS) score was correlated with higher conversation frequency during the day (9 AM-6 PM: the time frame participants might be in classes) and the evening (6 PM-0 AM), longer conversation duration during the day, and longer sleep duration. One study that evaluated self-reported depression using mobile phones for 2 weeks (N=28) [8] showed that mobility patterns (ie, regularity in 24-hour mobility patterns, as well as location variance) from Global Positioning System and phone usage features including usage duration and frequency were correlated with depressive symptom severity on a self-reported depression survey, the Patient Health Questionnaire-9 (PHQ-9) [8]. Another mobile phone–based study that lasted 12 weeks (N=73) identified mobile phone features that predicted clinically diagnosed depressed mood with 0.74 area under the curve; these features including the total count of outgoing calls, the count of unique numbers texted, absolute distance traveled, dynamic variation of the voice, speaking rate, and voice quality [10].

The combination of wearable sensor and mobile phone data has also been used to study self-reported stress in daily life [11-14]. Muaremi et al, using both wearable sensors and mobile phones, developed a way to automate the recognition of self-reported daily stress levels using sleep parameters and 37 physiological responses (including heart rate, heart rate variability (HRV) and SC) from wearable sensors (N=10, 19 days), or mobile phone usage and sleep HRV from wearable sensors (N=35, 4 months). They showed 61% 3-class stress level classification accuracy with a combination of phone usage and sleep HRV features and 73% accuracy using sleep duration, upper body posture, and sleep HRV features [11,12]. Sano et al also investigated 5-day self-reported high or low stress recognition (N=18) and 1-month high or low stress recognition (N=66) using wearable sensor and mobile phone data; they showed 75% and 90% accuracy using leave-one participant-out or 10-fold cross-validation, respectively [13,14].

### Objectives

These previous studies focused on only mobile phone usage or on phone usage plus wearable sensor data only during sleep and have not taken advantage of 24/7 multimodal phone + wearable data during wake and sleep to understand behaviors and physiology for long-term study of self-reported stress and mental health. We chose to approach this goal beginning with college students, most of whom report high stress, and some of whom are at risk of low or declining mental health [15,16]. According to the 2017 National College Health Assessment that examined data from 47,821 college students at 92 schools in the United States, more than half of the respondents said that their stress levels were higher than average, more than one-third had difficulty functioning because of depression, and two-thirds said they felt overwhelming anxiety in the last year [15]. Students’ high stress and low mental health could negatively impact their academic performance [17]. Moreover, one-tenth of the students had a plan for suicide. Suicide rate is increasing, and suicide is the second leading cause of death for college students [18]. More students are seeking help, and 34% of counseling centers have a treatment waitlist [19]. Under these conditions, development of improved tools for screening, monitoring, and intervening for self-reported stress and poor mental health through wearable sensors and mobile phones in daily life settings will be beneficial. We aim to ultimately detect stress and mental health changes before clinical interventions are required and provide personalized early warnings together with data-driven suggestions of individualized behaviors that might promote better mental health outcomes.

Our SNAPSHOT study was designed to collect and examine rich multimodal information in participants’ everyday life using wearable sensors and mobile phones for phenotyping sleep, stress, and mental health, all of which are major health issues in modern society. This paper has three main elements. First, we introduce a methodology and tools to capture long-term, large-scale ambulatory data on physiological and behavioral characteristics using sensors installed in wearable devices and mobile phones. The dataset from the SNAPSHOT study is one of the first large multimodal datasets that contains continuous physiology from a healthy college student population. The dataset currently includes approximately 145,000 hours of data from 201 participants at one university. Second, as real-world ambulatory data are messy, we describe tools we developed and deployed to improve the integrity and quality of the collected data and to reduce the time experimenters spend checking for and fixing errors. Third, we identify objective physiological markers and modifiable behaviors that successfully classify self-reported high or low stress and mental health and examine the separate contributions of wearable sensors and mobile phone data.

## Methods

The 1-month SNAPSHOT study is a long-term and large-scale study developed to measure Sleep, Networks, Affect, Performance, Stress, and Health using Objective Techniques. Our aim was to investigate how daily behaviors and social networks influence sleep, self-reported stress, mood, performance, and other well-being-related factors. For each of five Fall and Spring semesters starting in Fall 2013, we collected approximately 1 month of data per person from college students who were socially connected and at a single New England university. Students were only allowed to participate in the study once. There was a total of 201 participants; Fall 2013: 20, Spring 2014: 48, Fall 2014: 46, Spring 2015: 47, Fall 2015:40; ages 18 to 25 years; 129 male, 72 female; 70 freshman, 49 sophomore, 44 junior, 36 senior, and 2 unreported. The approximately 1 month of data collection was between the start of semester and midterms.

### Recruitment

We intentionally recruited college students from a single academic institution who were socially connected because of our interest in how social networks affect sleep and health behaviors. Our definition of socially connected was making a call or SMS at least once a week with each other. Each semester, we recruited groups of at least 5 people who knew each other and interacted socially. We posted our study advertisement to undergraduate students’ mailing lists. Potential participants filled out screening questionnaires to determine eligibility. Our exclusion criteria were as follows: (1) non-Android phone users, (2) inability to wear wrist sensors (eg, irritated skin on wrist), (3) pregnant women, (4) travel across more than one time zone 1 week before the study or have plans to travel more than one time zone away during the study, and (5) age <18 years or >60 years. In our study, we targeted only Android phone users because other mobile phones (eg, iPhone) did not allow us to monitor phone usage as needed for this study.

Eligible participants attended information and consent sessions. For each session, we invited approximately 15 participants and explained in detail the study and tasks that participants would perform during the study. After participants gave written informed consent, they completed prestudy questionnaires, started wearing devices, and installed an Android app (described below) on their phone. The study obtained a National Institutes of Health Certificate of Confidentiality so that potentially sensitive information such as drug or alcohol use provided by the participants could not be revealed for legal purposes; this was important protection for the students as the daily diary included requests for such information.

The participants received financial compensation at the end of the study; the amount depended on the number of days they completed diaries, wore the sensors, and completed other protocol tasks.

Study protocols were approved by the Massachusetts Institute of Technology and Partners HealthCare Institutional Review Boards. The study was registered on clinicaltrials.gov (NCT02846077).

### Data Collection

All data were deidentified before analysis, although location information could potentially be used to reidentify people. Phone numbers, email addresses, and actual names from the social network surveys were hashed.

#### Start of the Study Questionnaires

At the start of the study, participants completed the Morningness-Eveningness Questionnaire [20], the Pittsburgh Sleep Quality Index [21], the Myers Brigg Personality test, the Big Five Inventory Personality Test [22], the PSS [23], the 12-Item Short Form Health Survey (SF-12) for physical and mental component summary (MCS) scores [24], and a set of social network surveys assessing with whom participants spent their time to help map their social networks. We also collected age, sex, academic major, and living situation (eg, dorm name and whether single or multiple occupancy room) information.

#### Ambulatory Monitoring

##### Wearable Sensors

Participants wore two sensors on their wrists: a Q-sensor (Affectiva, Boston, MA, United States) to measure SC, skin temperature (ST), three-axis *acceleration* (ACC) on their dominant wrist and a Motion Logger (AMI, Ardsley, NY, United States) on their nondominant wrist to measure acceleration and ambient light data. ACC can be used to estimate activity levels and sleep or wake patterns. SC reflects autonomic arousal during the day, providing a stress index during wakefulness; SC increases during sleep are highly likely to occur in either non-rapid eye movement (non-REM) stage 2 sleep or slow-wave sleep (SWS) [25]. The sensor data were logged into the flash memory of the sensors. Participants were instructed to remove sensors only in instances when the sensor could become wet or risked being broken.

##### Mobile Phone App

We wrote a custom Android phone app based on funf [26] that monitored location, receivers, senders, and timings of calls and SMS text messages, screen on or off timings, and phone app usage. No content of emails, calls, or SMS text messages was recorded. Phone usage was measured for two main reasons. First, phone usage and location data give clues to social interactions. The timing of calls, SMS, and screen on provide an estimate of how often participants interact with their phone during the day and the night, whereas the number of calls, SMS, and the number of people they interact with helps quantify their social interaction. Second, lighting from the interaction with mobile phones or emailing late at night could disturb the biological circadian clock and increase alertness, both of which can influence sleep patterns [27,28]. We asked our participants not to use third-party messaging apps, if possible, during the study for the last two cohorts.

##### Twice-Daily Electronic Diaries

**Figure 1:**
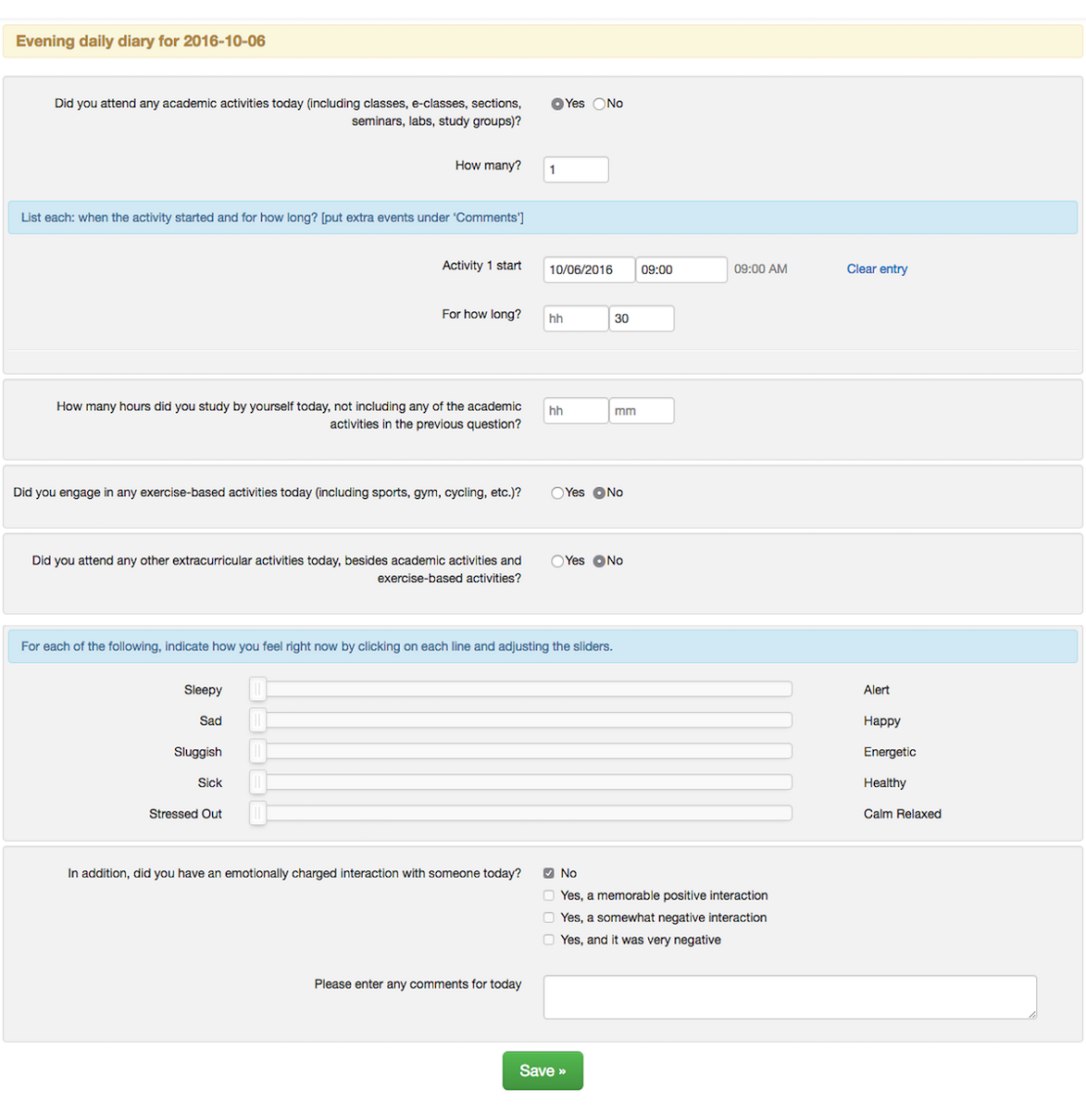
An example evening e-diary. For some questions, if yes is chosen, additional questions are presented.

Participants completed electronic diaries (e-diaries): upon awakening and at bedtime each day. These diaries contained questions about sleep and wake times; naps; exercise; academic and extracurricular activity times; social interactions; caffeine, alcohol, and drug intake; overall health condition; sleep; mood; and self-reported stress (Figure 1). Participants received emails that included a URL to the morning and evening diaries. They could complete the diaries using computers, tablets, or mobile phones.

##### Poststudy Questionnaires and Other Measurements

At the end of the month of intensive data collection:

1. Academic performance as measured by grade point average was self-reported by each participant for the semester previous to the study and the current study semester.
2. Email usage during the experiment (ie, to, from, cc, and time stamps) was collected through the Massachusetts Institute of Technology (MIT) website Immersion [29].
3. On the basis of their phone call, SMS, and email usage objectively measured during the experiment, participants were asked to self-report whether they had positive or neutral or negative interactions with each frequent contact as a whole over the month. Participants also indicated to which category each frequent contact belonged to (ie, family, social, work, others).
4. The PSS, the SF-12, the set of social network surveys, and the State-Trait Anxiety Index [30] were completed.

### Data Preprocessing

Ambulatory data measured with wearable sensors, mobile phones, and surveys tend to be noisy. Examples include (1) AM vs PM errors when participants complete survey items about their sleep and activity times; (2) participants forgetting to charge or wear sensors; (3) sensors breaking or the signals becoming noisy; and (4) mobile phone connectivity, hardware sensor functionality, and mobile software updates, which can break and interfere with data integrity. To address these issues, various techniques have been applied, such as data cleaning before data analysis [31]: data quality evaluation [32], detecting faulty data, noise reduction [33], and interpolating faulty or missing values [34,35]. To reduce the occurrence or impact of these issues, additional approaches can be used during ambulatory data collection. For example, during the study, an e-diary system can notify participants about potential inaccurate answers before they submit their answers, and a study investigator can check data quality of incoming data and provide feedback to the participants. For this study, we developed tools for improving the quality of the collected data and for supporting more efficient human checking and correcting of the phone, sensor, and e-diary data.

#### Preprocessing Twice-Daily Electronic Diaries

We collected a total of 6077 days of e-diary data. In the first year of the SNAPSHOT study, we set up an e-diary system that automatically sent surveys to our participants every morning and evening and then sent reminders if the participants did not complete the surveys within 12 hours. We implemented logic check functions on the system that prompted users to revise their answers if certain types of errors or missing answers were detected (eg, if two activity events overlapped, or if their reported wake time was earlier than their reported bedtime). During this first year, study investigators manually checked participants’ answers every 1 to 2 days and emailed them to revise their answers when errors were found.

**Figure 2:**
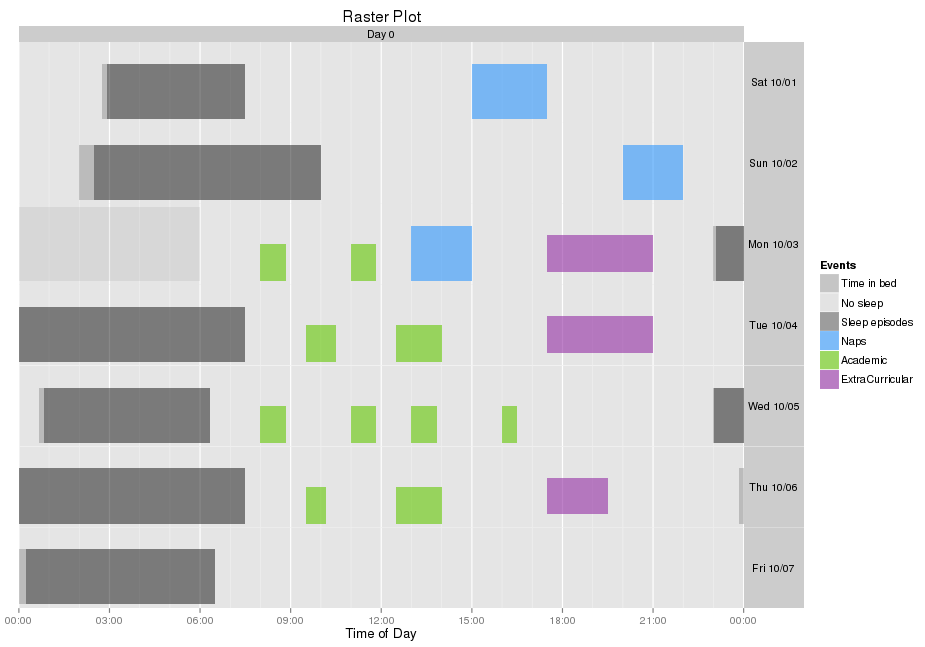
Plot of daily activity timing (raster plot) with time of day (midnight to midnight) on the y-axis and each day plotted on a separate line. Participants saw this plot after filling out their surveys and before they submitted their answers. Different activities were marked with different colors.

In year 2 of the study, we installed raster plots that visualize participants’ activities over time (Figure 2). These raster plots were displayed to participants after they submitted their answers, allowing users to visually confirm their responses and return to their survey to correct any errors. These raster plots reduced about half of the daily diary errors. The raster plots also reduced the total average time taken to preprocess 1 month of a participant’s e-diary data by 53%: from 145 min (year 1) to 68 min (year 2).

**Figure 3:**
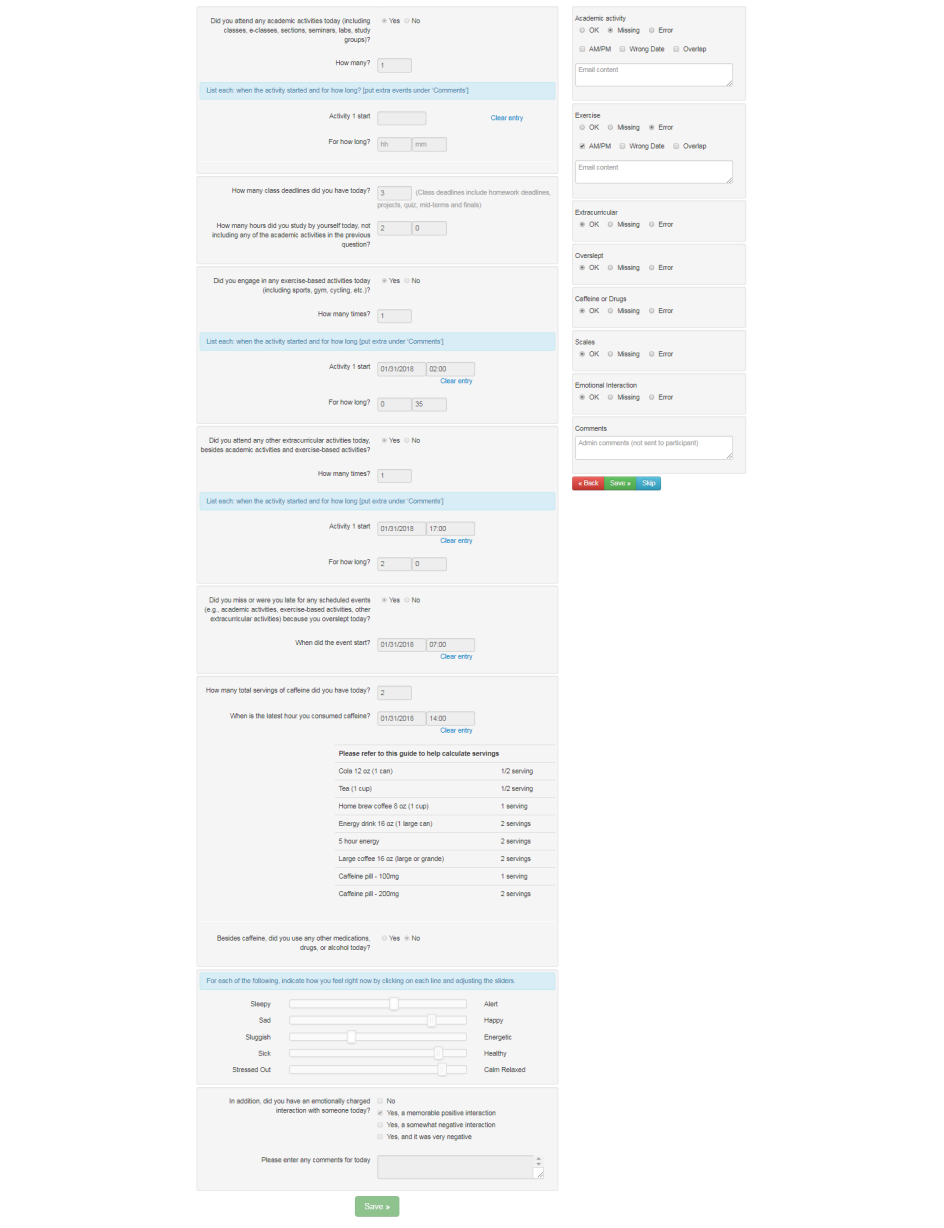
Interactive diary check system. The left panel shows a participant’s answers. The right panel shows if there are any detected errors or missing entries and enables adding comments. After the study investigator clicked the Save button, the system sent an email to a participant about any missing or erroneous entries if appropriate.

Finally, in year 3, we created and installed an administrative module that includes three components to further improve data validity: a calendar view, interactive checking system, and a summary view. Every day, a study investigator logged into the diary system and saw the calendar view (Multimedia Appendix 1) that showed the number of participants in the study, the number of participants whose morning and evening diaries were checked, the number of unchecked diaries, the number of diaries that needed to be rechecked, and participants’ comments. The interactive checking system automatically flagged missing answers in the e-diary and allowed the study investigator to check daily diaries just by flagging sections of the e-diary as error (Figure 3). Emails were automatically sent to participants if there were errors or missing answers. The summary view (Multimedia Appendix 2) showed the daily diary status for each participant in different colors (eg, green-acceptable, red-missing, and pink-error). These plots enabled the study investigator to understand which participants had filled out the daily surveys and which participants they needed to contact (eg, if there were repeated errors or missing entries in the diaries). This module further reduced the total average time taken to preprocess 1 month of a participant’s e-diary data from 68 min (year 2) to 45 min (year 3). The combined changes in raster plots and the administrative modules reduced the total average time taken to clean 1 month of one participant’s e-diary data by 69%: from 145 min (year 1) to 45 min (year 3). Overall, participants’ daily diary completion rates ranged between 92% and 97% with no significant differences across semesters.

#### Preprocessing Sensor or Mobile Phone Data

Every week, a study investigator had a face-to-face meeting with each participant to download sensor data and to check if sensors were working correctly, if the participants were wearing them properly, and if sensor electrodes needed replacement. We developed scripts to download the data from sensors and check sensor readings automatically for quality using a previously developed and tested automated classifier [36]. This classifier separated clean epochs and noisy epochs of SC data for further analysis.

We collected 6309 days of Q-sensor data for a total of 125,413 hours. We computed how much data were within a typical range per published guidelines: for SC, 83% were within the range of 0.01 to 30 microS [37-39], and for ST, 99.7% were within the range of 20 to 42 degrees Celsius [40]. In addition, 92% of the collected SC data were classified as clean data using an artifact detection algorithm [36]. Thus, among the collected SC data, 80% of the data were used for further analysis.

Mobile phone data were sent automatically to a server by the custom funf-based app. On the server, another set of scripts that we wrote checked the data quality every day and sent notification to a participant if a problem was found in their data (eg, not receiving phone data for a day). Phone data were collected on 85% of the days.

### Identifying Risk Factors, Objective Biomarkers, and Modifiable Behavioral Features Related to Stress and Mental Health

**Figure 4:**
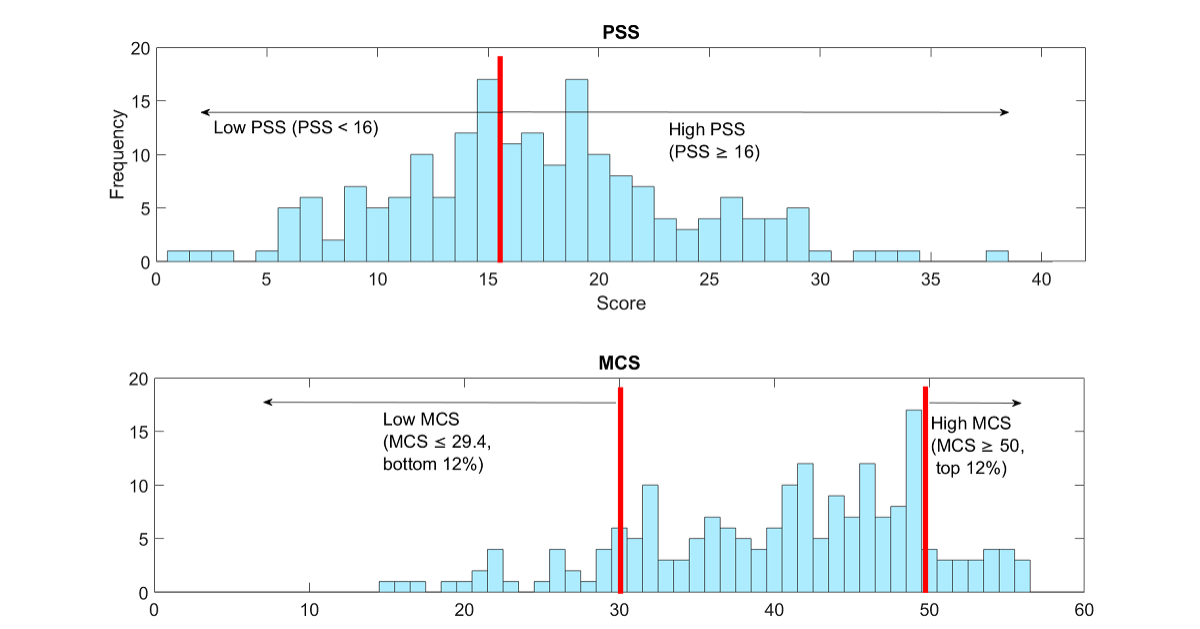
(1) Distribution of poststudy Perceived Stress Scale (PSS) and (2) Distribution of poststudy mental component summary (MCS) scores.

We defined high stress and low stress groups based on their poststudy PSS scores (Figure 4). PSS scores range from 0 to 40: higher scores indicate higher perceived stress. A PSS score of 14.2 is the average for the age group of 18 to 29 years, and a score over 16 is considered as high stress and of high health concern [23]. Our participants’ average PSS score was 17.1. We used the value of PSS ≥16 to construct the high stress group (N=109, top 57.7% [109/189]) and PSS <16 for the low stress group (N=80, bottom 42.3% [80/189]). Because we originally had an unbalanced set of data for high stress and for low stress, we first reduced the size of the high stress group by the method of random sampling of its data to equalize the size of the high and low stress classes at N=80. Thus, the prior probabilities on both classes were made to be 0.5, so that a random classifier would be expected to attain accuracy of 50%.

We defined high mental health and low mental health groups based on their poststudy MCS from the SF-12 (Figure 4). For the MCS, a value ≥50 is considered good mental health [41,42], and 11.8% (23/195) of our population scored ≥50. We therefore extracted the top and bottom 12% to form the two groups: high mental health group (MCS ≥50, top 11.8% [23/195], N=23) and low mental health group (MCS ≤29.4, bottom 12.3% [24/195], N=24). Thus, the data in the high and low mental health groups were balanced so that the prior probability of either group would be 0.5, with a random classifier expected to have an accuracy of 50%.

#### Feature Extraction

To quantify the relative importance of the many measures, we compared the classification performance using the following separate categories of features: (1) Big Five personality + gender, (2) wearable sensors (eg, ST, SC, and ACC), (3) mobile phone (eg, call, SMS, screen on, and location), and (4) objective features (combining wearable sensors and mobile phone metrics). We also separately defined (5) modifiable behaviors as features that can potentially be controlled by participants, such as sleep and activity timing and phone usage; these are important features to measure for future behavioral interventions (Table 1). Note that some features such as phone features and ACC feature are found in more than one of the five categories.

SC was processed first using low-pass filtering (cutoff frequency 0.4 Hz, 32nd order finite impulse response filter). Because there are individual differences in SC amplitude, we extracted features from both unnormalized and normalized SC data based on the maximum and minimum amplitude of each day within each individual. To detect SC peaks, we obtained the first derivative of the low-pass-filtered non-normalized SC data and then determined where the slope exceeded a value of 0.02 µS per second [43]. We detected SC peaks based on those that exceeded this threshold and counted the number of peaks in each 30-second epoch.

We used four different times of interest for analyses: day (9 AM-6 PM), night (6 PM-0 AM), late night (0 AM-3 AM), and sleep time (estimated for each individual from actigraphy and daily sleep diaries) as physiological responses, such as SC and ACC during daytime and sleep time have different meanings [44] and late night phone and exercise activities could relate to self-reported stress and mental health [45].

**Figure 5:**
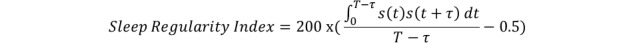
Equation of Sleep Regularity Index.

Bedtime and sleep regularity were calculated from the daily sleep diaries, and sleep duration and sleep efficiency were estimated from actigraphy with help of the daily sleep diaries. Sleep regularity was computed because a relationship between irregular sleep and low mental health was found in a previous study using this index [46]. The Sleep Regularity Index (SRI; Figure 5) captures the probability of an individual being in the same state (asleep vs awake) at any two time points 24 hours apart with 1 minute resolution, averaged across the entire study [47], where s(t)=1 during wake and s(t)=−1 during sleep for each minute. Assume data are collected for [0, T] with T=total number of hours of data and τ=24 hours.

In practice, individuals will only display sleep patterns that range between an SRI of 0 (random) and 100 (periodic: an individual who sleeps and wakes at exactly the same times each day). Values less than 0 are theoretically possible (eg, alternating 24 h of sleep and 24 h of wake) but very unlikely to be observed.

Phone usage and location data can provide information on sociability. We computed the timing and the number of calls, SMS, and screen on, which provide an estimate of how often participants interact with their phone during the day and the night. Previous studies showed the relationships between long phone usage duration and high stress [45] and long and frequent phone usage and severe depressive symptoms [8]. We also computed the number of people each participant interacted with over calls and SMS to help quantify their social interaction. For mobility features, we computed the distance and radius based on locations to which our participants travelled as these features were shown to be important in previous studies [8,48]. Additionally, because our population spent most of their time on campus or at their residence, we computed whether the day’s mobility pattern varied from the typical routine based on a Gaussian mixture model trained for each participant’s 1-month mobility patterns [49].

#### Classification

For classifying high or low stress groups and high or low mental health groups, we compared the methods of least absolute shrinkage and selection operator (LASSO), support vector machine (SVM) with linear kernel classifier, and SVM with radial basis function (RBF) kernel classifier; these algorithms were used in previous related work [8,10]. LASSO is a logistic regression that performs regularization and feature selection by minimizing the least squares objective function with an L1 penalty [50].

**Table 1:** List of features.

Modality	Features
All	Personality types, gender, diary, sensor, and phone features
Big Five personality types, gender (6 features)	Openness, conscientiousness, extraversion, agreeableness, neuroticism, gender
Sensors (17 features x 4 time frames x 3=204 features)	Mean, median, SD of 0 AM-3 AM, sleep, 9 AM-6 PM, 6 PM-0 AM for SC^a^, ACC^b^, and ST^c^
	Skin conductance: Area under the curve for 30 s epochs, max, mean, median, and SD of amplitude; mean, median and SD of peaks for 30 s epochs; mean, median, and SD of normalized amplitude
	Acceleration: total # of zero crossing for 30 s epochs
	Skin temperature: max, min, mean, median, and SD of temperature
Phone (25 features (call, SMS^d^, screen) x 3 time frames x 3 + 4 features (mobility) x 3 features=237 features)	Mean, median, SD of 0 AM-24 AM, 0 AM-3 AM, 6 PM-0 AM for call, SMS, and screen (not mobility)
	Call*:* Mean, median, and SD of duration and time stamp of calls per day*;* total duration per day, total number per day, and number of unique people per day
	SMS*:* Mean, median, and SD of duration and time stamp of SMS per day; total number per day and number of unique people per day
	Screen*:* Mean, median, and SD of screen-on duration and screen-on time stamp per day; total duration per day and total number of on or off per day
	Mobility: Total distance per day, 5-min distance, radius per day, and log likelihood of each day
Objective (441 features)	Phone and sensor features (see above)
Modifiable behaviors (296 features)	Sleep Regularity Index
	Mean, median, and SD of bedtime and sleep duration
	Diary features (see below)
	ACC total # of zero crossing for 30 s epochs
	Phone features (see above)
Diary (17 x 3=51 features)	Mean, median, SD of sleep or no sleep (pulled an all-nighter; binary valued), pre sleep electronic media interaction (emails, calls, SMS, Skype, chat, and online games; binary valued), pre sleep personal interaction(binary valued), # of naps, nap duration, # of academic activities per day, total academic duration, study duration, # of extracurricular activities, total extracurricular activities, # of exercise, exercise duration, # of caffeinated drink intake, memorable positive interaction(binary valued), somewhat negative interaction (binary valued), very negative interaction(binary valued), last caffeine intake time
Sleep (1 + 3 x 8=25 features)	Sleep Regularity Index
	Mean, median, and SD of bedtime, sleep duration, sleep efficiency, sleep or no sleep (pulled an all-nighter; binary valued), pre sleep electronic media interaction (emails, calls, SMS, Skype, chat, and online games; binary valued), pre sleep personal interaction (binary valued), # of naps and nap duration

^a^SC: skin conductance.

^b^ACC: acceleration.

^c^ST: skin temperature.

^d^SMS: short message service.

For training and testing models, we used nested-cross validation. To evaluate model performance, we applied leave-one-cohort-out: training a model with all except one semester cohort’s data and testing the model against the left-out cohort’s data, repeating this process for the total number of cohorts (ie, 5 times). First we (1) split the data into two datasets: a training set made up of four cohorts and a test set made up of one cohort. We then left the test set out until step (5) or (8) below.

For training the SVM models, we applied sequential forward feature selection to the training data to reduce overfitting and find the best combinations. (2) We applied a *t* test to each feature of the training datasets and selected 100 features with the lowest *P* values for finding features to separate two groups effectively then (3) applied sequential forward feature selection [51]: applied an SVM RBF classifier with 10-fold cross validation to find the best up to five combinations from these 100 features and optimized hyperparameters (C for SVM linear and C and gamma for SVM RBF). Then, (4) we trained the SVM linear or RBF models with the selected features of the training data, (5) tested the models against the test data, and (6) repeated this process (1-6) five times.

For LASSO, (7) the penalization parameter was determined with the training data by 10-fold cross validation and (8) the trained model was tested using the test data. This process (1, 7, and 8) was repeated five times.

We computed overall accuracy and F1 scores by concatenating the five-cohort predicted output to compare the performance of the models to reduce the bias from splitting [52]. We computed 95% confidence levels using adjusted Wald test [53]. The F1 score is a measure of performance computed using precision (also known as positive predictive value) and recall (also known as sensitivity) as described in Equation 1, where precision is the number of correct positive results divided by the number of all positive results, and recall is the number of correct positive results divided by the number of positive results that should have been returned.

(1) F1 = 2 x precision x recall / (precision + recall)

We also compared the performance of the models using features based on data from the entire 1-month study period with that using features based only on using the data from the week before the PSS and MCS surveys were completed.

We applied *t* tests or Mann-Whitney *U* tests (for non-Gaussian distributions) to examine if the means of the features were statistically different between the high or low PSS groups or the high or low MCS groups. We adjusted for the multiple comparisons using false discovery rate (FDR).

## Results

### Relationships Among Prestudy and Poststudy Perceived Stress Scores and Mental Component Summary

There were no differences in the poststudy PSS or MCS among the five cohorts; one-way analysis of variance (*P*=.20, F=1.50). Students’ poststudy scores (both PSS and MCS) were highly correlated with prestudy scores (*r*=.59, .60, Pearson correlation). Poststudy PSS scores statistically increased (mean prestudy PSS: 15.0, poststudy PSS: 17.1, paired *t* test, *P*<.001) and MCS scores decreased compared with the prestudy scores (mean prestudy MCS: 44.4, poststudy MCS: 40.4, Wilcoxon signed rank test, *P*<.001). Thus, the students reported worsening stress and mental health over the 1 month of measurement.

The poststudy PSS was inversely correlated with the poststudy MCS (*r*=−.71, Pearson correlation; Multimedia Appendix 3): (1) 83% (19/23) of the students in the high MCS group belonged to the low PSS group and (2) 88% (21/24) of the students in the low MCS group belonged to the high PSS group. The low MCS group had higher PSS scores than the rest of the students in the high PSS group: low MCS group’s average PSS score was 25.2, whereas the rest in the high PSS group’s average PSS score was 20.7 (*P*<.001).

### Stress and Mental Health Classification

**Figure 6:**
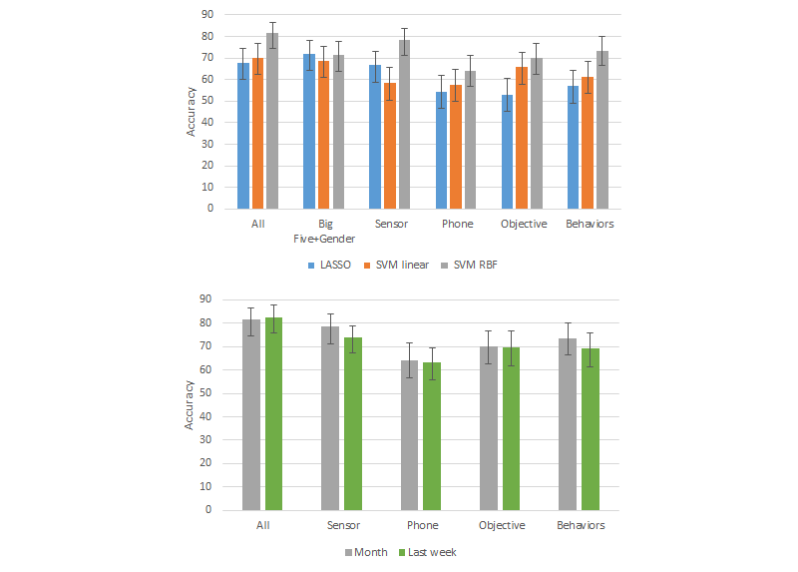
High or low Perceived Stress Scale (PSS) classification results. Top: comparison of performance using 1 month of data with three machine learning algorithms. Bottom: comparison of performance using 1 month of data vs only the last week of data with support vector machine radial basis function (SVM RBF). Accuracy scores for Big Five + Gender data are not shown in the bottom graph because these data are collected only once. Error bars indicate the 95% CIs based on adjusted Wald test.

**Figure 7:**
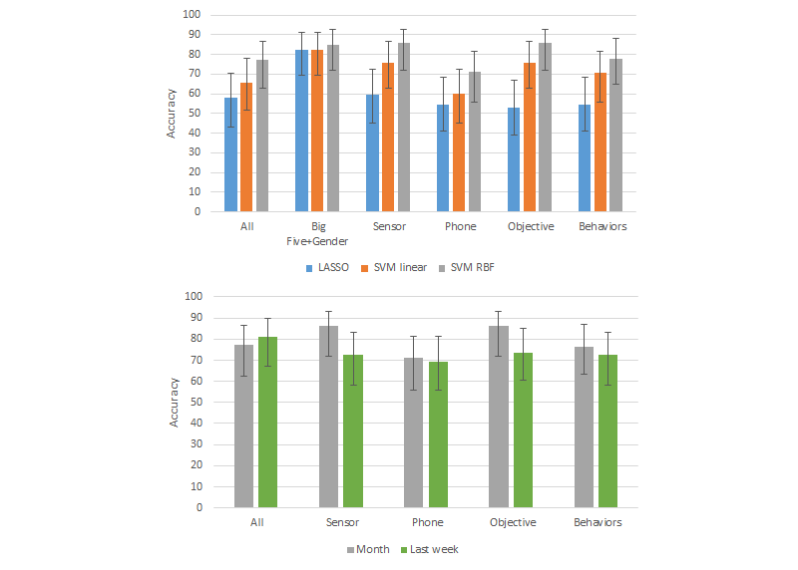
As in Figure 6 with high or low mental component summary score classification results, accuracy scores for Big Five + Gender data are not shown in the bottom graph because these data are collected only once. Error bars indicate the 95% CIs based on adjusted Wald test.

Overall, we found SVM models with the RBF kernel worked better than LASSO and linear SVM models using RBF kernels for all of the metrics (Figures 6 and 7; see Multimedia Appendix 4 for accuracy and F1 scores and Multimedia Appendices 5 and 6 for F1 scores for all results). SVM with the RBF kernel can model more complex decision boundaries. Sensor features showed higher performance than phone features both for PSS and MCS.

We also compared the performance of the SVM RBF models using features from only the last week of the 1-month period to using the features from the entire month. Overall, the performances with the 1 month of features were better (classification accuracy improved by 1-16%) than those using just the last week of features, except in the case of the SVM models using all features.

The accuracy for PSS classification was highest when using all features (82%), followed by when using features from only sensors (78%), only behaviors (74%), only the Big Five (71%), or only objective data (70%). The same rank ordering also held when comparing F1 scores. For MCS, sensor features and objective features showed the highest accuracy (87%), followed by Big Five (85%), behaviors (79%), and all (77%). The ranking of the F1 scores was similar except for all features had a slightly higher F1 than behaviors. The means and SD of the accuracy and F1 scores from leave-one-cohort-out cross validation are presented in Multimedia Appendix 7.

We also tested different cutoffs: (1) instead of PSS cutoffs ≥16 for high and <16 for low stress, we used PSS ≥14 for high stress group and PSS <14 for low stress, as (as noted above) 14.2 is the reported average for people aged 18 to 29 years [23] and (2) instead of extreme MCS cutoffs (top and bottom 12%), we used MCS ≥median (42.05) for high mental health group and MCS <median for low mental health group). This was done to test if the rankings of performances were sensitive to the exact cutoff values. Sensor and modifiable behavior features worked best with both cutoff values (Multimedia Appendices 8 and 9). Compared with the extreme MCS cutoffs, the median cutoff showed much lower classification performance (the accuracy decreased by 21 to 6 %).

**Figure 8:**
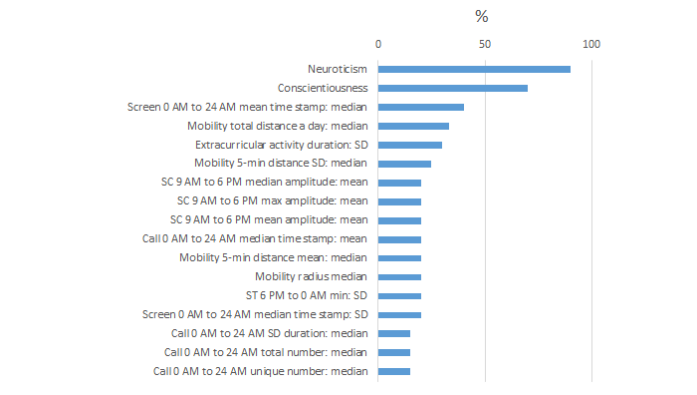
Percentage of time each feature was selected across 10-cross-validation for high or low Perceived Stress Scale (PSS) classification models with 1 month of data.

**Figure 9:**
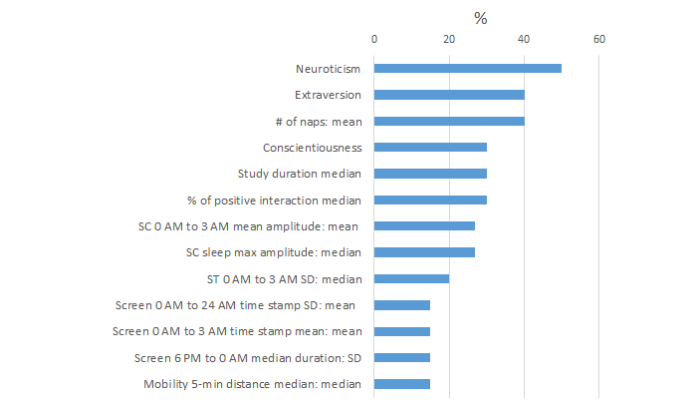
Percentage of times each feature was selected across 10-cross-validation for high or low mental component summary (MCS) classification models with 1 month of data.

We summarize the features most commonly selected by the algorithms as useful for high or low PSS detection (Figure 8) and high or low MCS detection (Figure 9) using the full 1 month of data. Percentages indicate the percent time these features are selected across 10-fold cross validation over five cohorts and five feature modalities (all, Big Five + gender, sensor, phone, objective and modifiable behavior features).

For PSS classification for self-reported stress, neuroticism and conscientiousness were the most often selected features (90% and 70% of the models, respectively). The high PSS group had higher neuroticism (q [which is the FDR-adjusted *P* value]=.0004). The high stress group had a larger extracurricular activity duration SD (q=.04).

In the MCS classification for self-reported mental health, the low MCS group showed higher neuroticism (q<.001) and lower conscientiousness (q=.04) than the high MCS group. The low MCS group had naps more frequently (40%; q=.04). In the MCS classification models using only the last week of data, the low MCS group showed a lower probability of interacting with electronic media (eg, emails, calls, SMS, Skype, chat, and online games) before sleep (30%; q=.004) and lower SD of the number of SC peaks during the time frame of 0 AM to 3 AM (20%; q=.03), as well as higher neuroticism (q<.001).

The percentages of time each feature was selected for each fold of leave-one-cohort cross validation are presented in Multimedia Appendices 10 and 11.

We also tried building models only with sleep features (eg, features in the sleep category and some sleep related features in the survey category). We obtained 72% and 65% accuracy for classifying high or low PSS and high or low MCS. Mean nap duration was the most common feature used for the PSS models (80% of the models), followed by median bed time and the frequency of pulling all-nighters (60%). The frequency of pulling all-nighters (100% of the models), mean number of naps, sleep duration, and sleep efficiency (60%) were commonly selected features by the MCS classification models. Average sleep duration was not significantly different statistically in the high vs low PSS groups or in the high vs low MCS groups (high PSS: 6 hours 42 min vs low PSS: 6 hours 51 min [*P*=.09], high MCS: 6 hours 40 min, low MCS: 6 hours 34 min [*P*=.72]). Instead, the low MCS group’s more frequent napping was one of the most discriminating features.

## Discussion

### Principal Findings

In this paper, we developed novel tools to collect and process objective physiological and behavioral measures using online diaries, wearable sensors, and mobile phones. We aimed to investigate how accurately these measures could identify conditions of self-reported high stress and poor mental health and features most accurate in identifying these conditions.

Physiological sensor, phone, and mobility features were the best predictors for distinguishing self-reported high or low stress and mental health. Wearable sensor features, including SC and ST, reached 79% accuracy for classifying high or low stress groups and 87% accuracy for classifying high or low mental health groups. Modifiable behaviors, including number of naps, studying duration, phone calls (number, time stamp and duration of calls), mobility patterns, and phone-screen-on time, reached 74% accuracy for high or low stress group classification and 78% accuracy for high or low mental health group classification.

### Comparison With Prior Work and Interpretations of Our Results

Our analysis showed that relatively high accuracy and F1 scores can be achieved using the leave-one-semester-cohort-out testing of the machine learning classifier for high or low stress measured by PSS and high or low mental health measured by MCS. Of all the features tested, the sensor features resulted in approximately 14% higher classification accuracies in both PSS and MCS than the phone features. In particular, SC responses during the time frame of 9 AM to 6 PM were one of the best predictors for PSS. SC has been considered as a biomarker for stress [44] because SC quantifies eccrine sweat activity that is controlled by only sympathetic nervous activity. These findings (1) are among the first to show the potential contribution of SC in stress detection using a wrist wearable sensor in a 24/7 daily life setting and (2) agree with previous findings that use a conventional finger SC sensor or a wearable SC sensor in settings where a person is seated, eg, driving a car. For example, Healey et al measured SC, heart rate, HRV, respiration, and electromyogram in Boston drivers and reported that SC was the most associated with stress [54]. Additionally, Hernandez et al discriminated stressful and nonstressful calls at a call center environment using SC features with 78% accuracy [55], and Setz et al automatically classified SC responses from cognitive load and stress with accuracy higher than 80% [56].

As we examined more closely which sensor features were most discriminative, we found that SC responses during the time frame of 0 AM to 3 AM and during sleep were predictors for separating high and low self-reported mental health. Some studies have shown that finger-based SC are reduced for patients with depression measured in a short-term lab study [57-59]. One possible explanation of how low SC responses during sleep could be related to MCS scores is that there is a decrease in SWS in depression [60] and other psychiatric disorders [61], and the largest SC responses during sleep are likely to occur during non-REM stage 2 and SWS [25]. Note that in our data, (1) 0 AM to 3 AM could include both awake and asleep conditions, and if it included sleep, we would expect it to include more SWS being at the start of the night for this cohort; (2) our low mental score groups are based on self-report; and (3) we do not know if any of our participants had clinically defined depression or other psychiatric disorders as that information was not gathered as part of this study.

We found that ST features were also predictors for PSS and MCS. A previous study has shown that acute stress does reduce distal finger ST but does not statistically significantly reduce wrist ST in a laboratory stress test setting [62]. Furthermore, another study showed that ST is one of the strongest discriminants to distinguish sleep and wake states [63]. Another study showed that patients with depression have less rhythmicity in ST [64], which would also be consistent with less regular sleep in depression. To our knowledge, this paper is the first to report that ambulatory wrist ST features are related to self-reported stress.

For phone features, our results showed phone usage time stamp and duration can be predictors for PSS and MCS. These results are consistent with several previous studies. People with a PHQ-9 score higher than 5 showed longer phone usage and higher phone usage frequency than those with a PHQ-9 score lower than 5 in a 2-week study with mobile phones [8]. A questionnaire-based study also showed a relationship between high mobile phone usage, stress, and symptoms of depression [45].

Mobility, specifically travel distance per day and SD of the distance traveled as measured by phone geolocation data, is a predictor both for self-reported stress and mental health. This result agrees with one study [8] reporting that normalized mobility entropy (distribution of frequency of visiting different places) and location variance were negatively correlated with depression symptoms and another study reporting that mobility patterns were highly related to stress level [13]. The relationship between reduced activity levels and mobility patterns and high stress and low mental health has been studied [48,65]. These behavioral markers could be an objective index for monitoring self-reported low mental health. It is possible that encouraging people to move more could be an effective intervention to reduce stress and improve mental health.

Consistent with previous studies [7,13,14], personality types were one of the most influential and statistically significant factors for self-reported stress and mental health in this college population. In the Big Five Inventory Personality Test categories, neuroticism was a predictor of stress [66]. The combination of low extraversion and low conscientiousness or low agreeableness contributed to the high stress group; these directions of the associations in our analysis were consistent with prior work [67]. High neuroticism and low extraversion have previously been associated with low MCS [68].

There is a known association between sleep deficiency and mental health status (eg, [61]). Our results, however, did not show that sleep duration was a strong discriminant feature for self-reported stress and mental health.

### Limitations

There are multiple limitations of this study:

1. Selection of a feature as discriminating between two categories does not mean it is an important feature or causative of that behavior.
2. These results do not tell us the causality (eg, does a student sleep later and less regularly because of higher stress or have higher stress because of later or more irregular sleep?).
3. Our participants were limited to Android phone users because we wanted to log detailed phone usage, which is not allowed by other phone systems such as iPhone. As about half of the undergraduate students were Android users on the campus, a selection bias might exist. A previous study showed slight differences in personality types and economic status between iPhone users and Android users [69].
4. A total of 64% of our study population were male participants. It has been reported that females report higher perceived stress levels and more depressive symptoms [70-73], and there are gender differences in psychological and biological stress responses [74]. In our dataset, the ratios of female participants in the high or low PSS and MCS groups were 45% and 20% (high and low PSS) and 22% and 54% (high and low MCS). Modeling stress and mental health differently in males and females might help understand the mechanism. Gender was included as a potential feature in our models and was not selected frequently.
5. Our data come from college students at one New England university over 4 years. The work needs to be applied to other populations to determine generalizability.
6. Our data come from socially connected student groups. We might observe some statistically coherent behaviors in our dataset because of these connections.

### Future Work

These new tools and methods can allow multimodal data in daily life to be captured more continuously, with greater accuracy and integrity of the data, and for long-term and at great scale. We are planning to collect a larger amount of data for an even longer time to study long-term behaviors and physiological responses and build predictive models. To do this, we need to build a new system for consenting people in remote locations, fully automate checking their measurement status and data accuracy automatically, and let the participants know about errors so they can fix them to keep study compliance and data accuracy high.

We will continue our data analysis for understanding behaviors, physiological responses, and traits that impact health and well-being. One of our hypotheses is that health-related behaviors will be contagious within social networks and that social network data we obtained from call, SMS, and email data could capture the social contagion quantitatively instead of requiring self-report to capture it. We are also interested in studying how phone usage influences sleep and health and how we can predict stress and mental health using previous behaviors and physiology.

These machine learning models are not limited to modalities and features we measured and computed in this study but can also be used for other modalities such as heart rate and heart rate variability that are controlled by autonomous activities, and other features such as app usage, ambient light, and audio or sentiment-based patterns extracted from text or speech could be added to improve the models. The features and models presented in this paper can be tested in similar multimodal ambulatory datasets collected in other future studies. Tracking stress and mental health conditions would help students better understand their stress and mental health conditions over multiple semesters, as well as help clinicians see how treatment affects students’ conditions if they receive treatment.

### Conclusions

In this paper, we introduced a methodology and tools we developed to measure ambulatory multimodal data and improve the integrity of collected data to study self-reported stress and mental health in the daily lives of college students. We showed that objective and modifiable behavioral features collected over 1 month can classify these college students as high or low stress based on the PSS and as having high or low mental health based on MCS from SF-12 collected at the end of that month with over 70% accuracy, whereas sensor features alone could classify high or low mental health and achieve over 88% on an F1 score. For classifying high or low stress groups, we found that combining phone and sensor features typically gave the best results over using either modality alone, whereas for classifying high or low mental health groups, the use of wearable sensor features performed comparable to wearable + phone features.

## Appendix

Multimedia Appendix 1 Calendar view. For each day, the investigator can see how many participants are in the study, how many surveys have been verified, how many need to be re-examined, and participant comments.

Multimedia Appendix 2 Summary view. The investigator can see the daily diary status for each participant in different colors: green: acceptable; red: missing; pink: error; red/white slash: missing but participants still have time to complete the diary.

Multimedia Appendix 3 The relationship between poststudy Perceived Stress Scale (PSS) and poststudy Mental Component Score (MCS) for each participant (blue circle). Chosen cutoffs for high and low PSS and high and low MCS are indicated.

Multimedia Appendix 4 Performance of PSS and MCS classification models with 1 month or last week of data.

Multimedia Appendix 5 High or low Perceived Stress Scale (PSS) classification results. Top: Comparison of F1 scores for PSS classification with three machine learning algorithms (LASSO, SVM linear, and SVM RBF) on 1 month of different types of data (All, Big Five + Gender, Sensor, Phone, Objective, Behaviors). Bottom: Comparison of F1 scores with SVM RBF machine learning algorithm on 1 month of data versus on only the last week of the same data types. Accuracy scores for Big Five + Gender data are not shown in the bottom graph because these data are collected only once.

Multimedia Appendix 6 High or low Mental Component Score (MCS) classification results. Top: Comparison of F1 scores for MCS classification with three machine learning algorithms (LASSO, SVM linear, and SVM RBF) on 1 month of different types of data (All, Big Five + Gender, Sensor, Phone, Objective, Behaviors). Bottom: Comparison of F1 scores with SVM RBF machine learning algorithm on 1 month of data versus on only the last week of the same data types. Accuracy scores for Big Five + Gender data are not shown in the bottom graph because these data are collected only once.

Multimedia Appendix 7 Mean and SD of accuracy and F1 scores from leave-one-cohort-out PSS and MCS classification models with 1 month of data and SVM RBF.

Multimedia Appendix 8 High or low Perceived Stress Scale (PSS) and Mental Component Score (MCS) classification results. Comparison of F1 for PSS and MCS classification scores with SVM RBF and 1 month of different types of data (All, Big Five + Gender, Sensor, Phone, Objective, Behaviors). Cutoff: 14 (the average in the 18-29 years age group) for PSS and 42.05 (median) for MCS.

Multimedia Appendix 9 Performance of PSS and MCS classification models with 1 month of data and SVM RBF. PSS cutoff: 14 (the average in the 18-29 years age group) and MCS cutoff: 42.05 (median).

Multimedia Appendix 10 Percentages of the number of times each feature was selected for each fold of leave-one-cohort-out cross validation for 1-month PSS models.

Multimedia Appendix 11 Percentages of the number of times each feature was selected for each fold of leave-one-cohort-out cross validation for 1-month MCS models.

## Abbreviations

ACC: acceleration
E-diary: electronic diary
FDR: false discovery rate
HRV: heart rate variability
LASSO: least absolute shrinkage and selection operator
MCS: mental component summary
MIT: Massachusetts Institute of Technology
PHQ-9: patient health questionnaire-9
PSS: perceived stress scores
RBF: radial basis function
REM: rapid eye movement
SC: skin conductance
SF-12: 12-Item Short Form Health Survey
SMS: short message service
SRI: Sleep Regularity Index
ST: skin temperature
SVM: support vector machine
SWS: slow-wave sleep
