# Supplementary figures and images for: Identifying Objective Physiological Markers and Modifiable Behaviors for Self-Reported Stress and Mental Health Status Using Wearable Sensors and Mobile Phones: Observational Study
Source: J Med Internet Res. 2018 Jun 8;20(6):e210. doi: 10.2196/jmir.9410 (PMC6015266; doi:10.2196/jmir.9410)

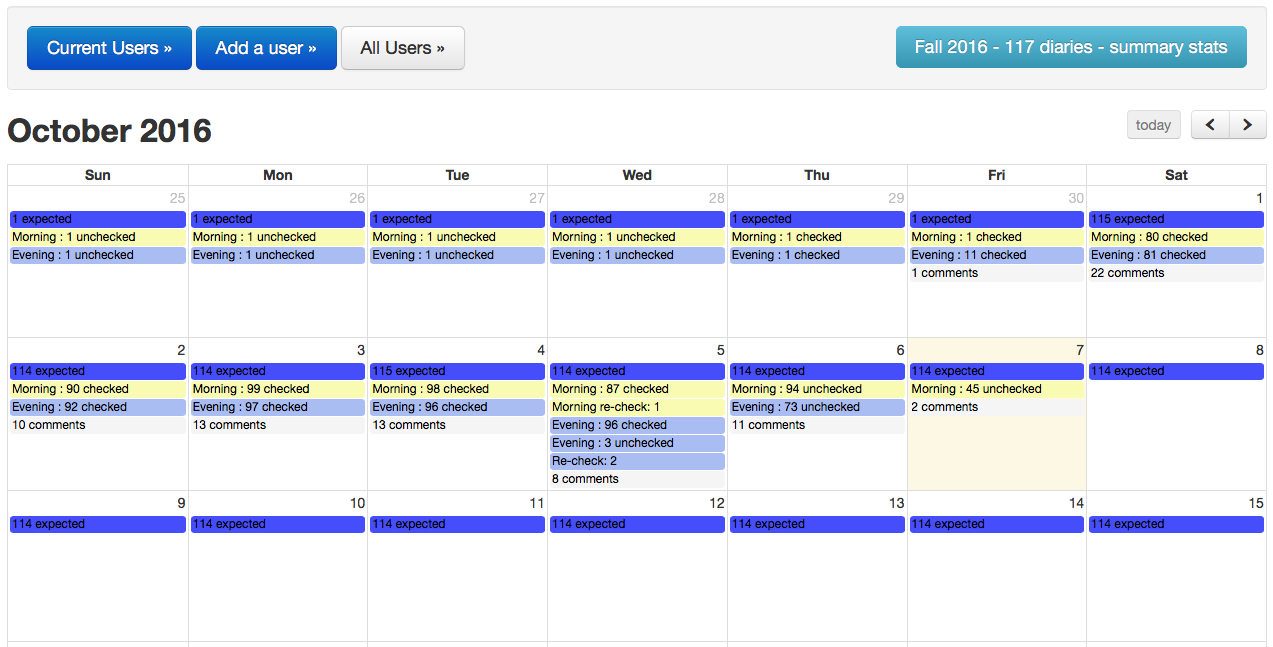

Supplement: Multimedia Appendix 1 [file jmir_v20i6e210_app1.png]

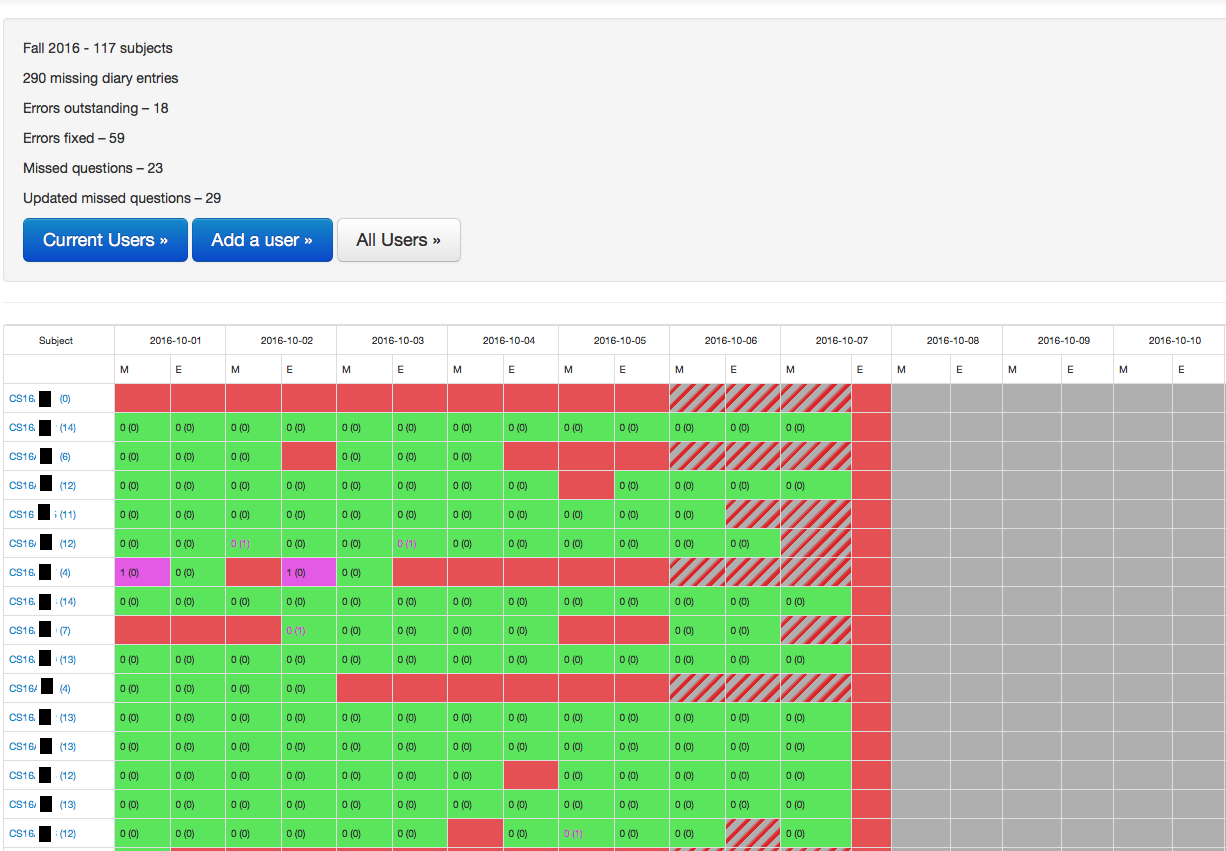

Supplement: Multimedia Appendix 2 [file jmir_v20i6e210_app2.png]

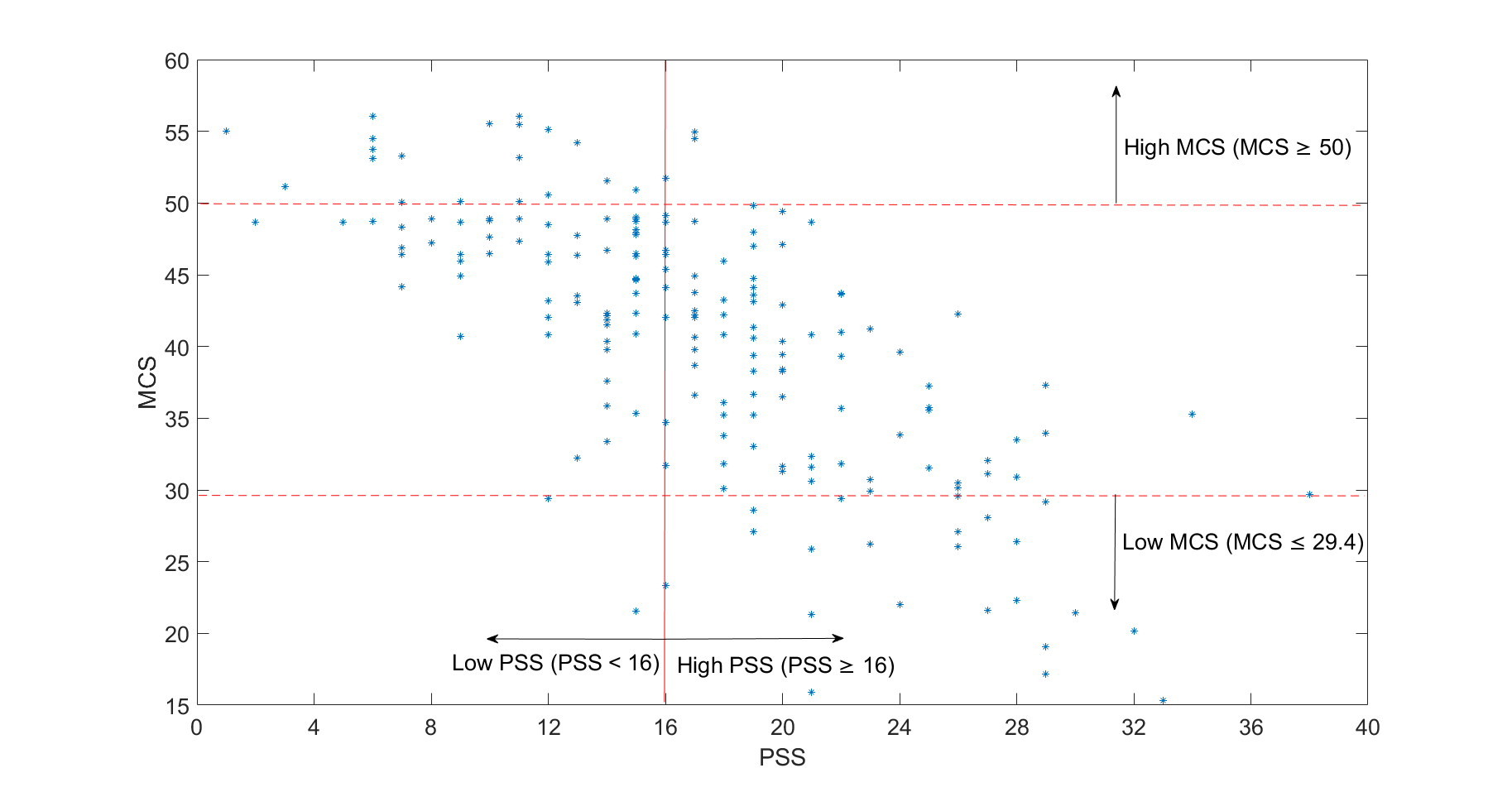

Supplement: Multimedia Appendix 3 [file jmir_v20i6e210_app3.png]

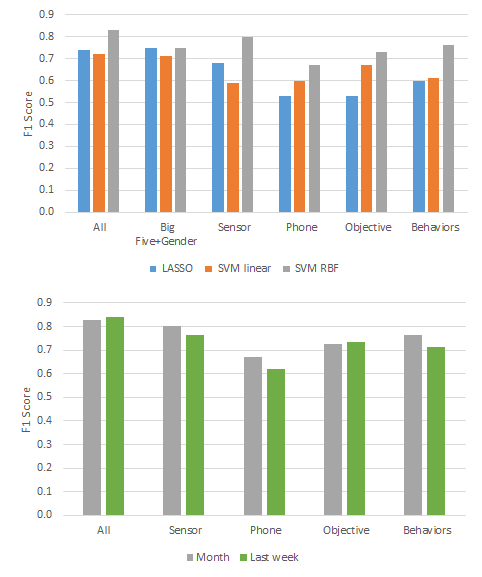

Supplement: Multimedia Appendix 5 [file jmir_v20i6e210_app5.png]

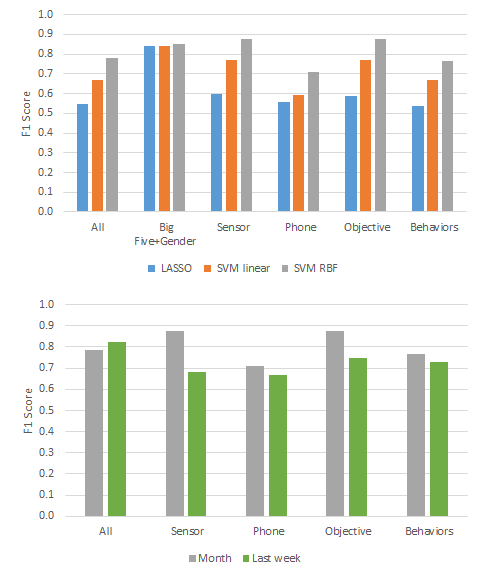

Supplement: Multimedia Appendix 6 [file jmir_v20i6e210_app6.png]

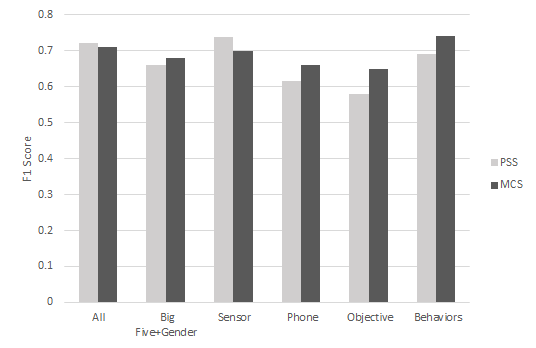

Supplement: Multimedia Appendix 8 [file jmir_v20i6e210_app8.png]
