# Supplementary material for: Identifying Objective Physiological Markers and Modifiable Behaviors for Self-Reported Stress and Mental Health Status Using Wearable Sensors and Mobile Phones: Observational Study
Source: J Med Internet Res. 2018 Jun 8;20(6):e210. doi: 10.2196/jmir.9410 (PMC6015266; doi:10.2196/jmir.9410)
Supplement: Multimedia Appendix 4 [file jmir_v20i6e210_app4.pdf]

Multimedia Appendix 4. Performance of PSS and MCS classification models with one month or last week of data.

	PSS (high or low)				MCS (high or low)			
	One month		Last week		One month		Last week	
	Accuracy	F1	Accuracy	F1	Accuracy	F1	Accuracy	F1
**All**								
LASSO	67.6	0.74	76.3	0.78	58.1	0.55	37.5	0.17
SVM linear	70.3	0.72	65.0	0.70	65.8	0.67	75.0	0.75
SVM RBF	81.5	0.83	82.5	0.84	77.2	0.78	81.3	0.82
**Big Five + Gender**								
LASSO	71.9	0.75	71.9	0.75	82.6	0.84	82.6	0.84
SVM linear	68.8	0.71	68.8	0.71	82.6	0.84	82.6	0.84
SVM RBF	71.4	0.75	71.4	0.75	84.7	0.85	84.7	0.85
**Sensor**								
LASSO	56.8	0.68	59.6	0.73	59.4	0.60	45.0	0.27
SVM linear	58.3	0.59	63.4	0.67	75.9	0.77	67.5	0.68
SVM RBF	78.3	0.80	73.8	0.76	85.1	0.88	70.0	0.70
**Phone**								
LASSO	44.1	0.53	39.6	0.39	54.4	0.56	47.1	0.47
SVM linear	57.5	0.60	47.9	0.57	60.0	0.59	64.7	0.60
SVM RBF	64.2	0.67	63.2	0.62	71.1	0.71	73.5	0.73
**Objective**								
LASSO	53.0	0.63	53.9	0.67	53.0	0.59	50.0	0.29
SVM linear	65.9	0.67	53.0	0.61	75.9	0.77	70.0	0.71
SVM RBF	70.1	0.73	69.6	0.73	86.1	0.88	73.3	0.75
**Behaviors**								
LASSO	46.9	0.60	37.2	0.35	54.7	0.54	68.8	0.74
SVM linear	61.2	0.61	59.0	0.61	70.6	0.67	81.3	0.84
SVM RBF	73.5	0.76	69.2	0.71	78.7	0.76	87.5	0.89
