# Supplementary material for: Identifying Objective Physiological Markers and Modifiable Behaviors for Self-Reported Stress and Mental Health Status Using Wearable Sensors and Mobile Phones: Observational Study
Source: J Med Internet Res. 2018 Jun 8;20(6):e210. doi: 10.2196/jmir.9410 (PMC6015266; doi:10.2196/jmir.9410)
Supplement: Multimedia Appendix 7 [file jmir_v20i6e210_app7.pdf]

Mean and standard deviation of accuracy and F1 scores from leave-one-cohort-out PSS and MCS classification models with one month of data and SVM RBF.

			All	Big Five+Gender	Sensor	Phone	Objective	Behaviors
**Accuracy**								
	PSS							
		Mean	78.5	75.2	82.7	77.9	75.0	80.7
		SD	16.8	14.1	14.4	14.1	9.3	14.1
	MCS							
		Mean	77.8	91.0	88.8	77.9	88.8	76.8
		SD	16.1	6.0	14.1	31.6	14.1	23.7
**F1**								
	PSS							
		Mean	0.84	0.80	0.83	0.78	0.75	0.77
		SD	0.11	0.12	0.06	0.07	0.10	0.10
	MCS							
		Mean	0.78	0.92	0.90	0.79	0.90	0.74
		SD	0.18	0.05	0.14	0.29	0.14	0.29
