# Supplementary material for: Identifying Objective Physiological Markers and Modifiable Behaviors for Self-Reported Stress and Mental Health Status Using Wearable Sensors and Mobile Phones: Observational Study
Source: J Med Internet Res. 2018 Jun 8;20(6):e210. doi: 10.2196/jmir.9410 (PMC6015266; doi:10.2196/jmir.9410)
Supplement: Multimedia Appendix 9 [file jmir_v20i6e210_app9.pdf]

Performance of PSS and MCS classification models with one month of data and SVM RBF. PSS cutoff: 14 (the average in the age group of 18-29 years old) and MCS cutoff: 42.05 (median).

	PSS (high or low)		MCS (high or low)	
	Accuracy	F1	Accuracy	F1
All	71.3	0.72	69.9	0.71
Big Five + Gender	64.4	0.66	63.4	0.68
Sensor	70.7	0.74	70.1	0.70
Phone	61.8	0.62	63.4	0.66
Objective	57.0	0.58	63.0	0.65
Behaviors	69.2	0.69	71.7	0.74
