# Supplementary material for: Identifying Objective Physiological Markers and Modifiable Behaviors for Self-Reported Stress and Mental Health Status Using Wearable Sensors and Mobile Phones: Observational Study
Source: J Med Internet Res. 2018 Jun 8;20(6):e210. doi: 10.2196/jmir.9410 (PMC6015266; doi:10.2196/jmir.9410)
Supplement: Multimedia Appendix 10 [file jmir_v20i6e210_app10.pdf]

Percentages of time each feature was selected for each fold of leave-one-cohort-out cross validation for one month PSS models.

Features used for PSS model (one month)	Leave-one-cohort-out cross validation folds				
	1	2	3	4	5
Neuroticism	100	100	100	100	50
Conscientiousness	50	100	50	50	100
Screen 0 AM to 24 AM mean time stamp: median	67	0	0	33	100
Mobility total distance a day: median	25	25	0	50	25
Extracurricular activity duration: SD	50	0	50	50	0
Mobility 5-min distance SD: median	25	0	25	25	50
SC 9 AM to 6 PM median amplitude: mean	0	33	33	0	33
SC 9 AM to 6 PM max amplitude: mean	0	0	33	0	67
SC 9 AM to 6 PM mean amplitude: mean	0	0	0	0	100
Call 0 AM to 24 AM median time stamp: mean	0	0	50	25	25
Mobility 5-min distance mean: median	25	0	0	50	25
Mobility radius median	25	0	0	25	50
ST 6 PM to 0 AM min: SD	33	0	0	67	33
Screen 0 AM to 24 AM median time stamp: SD	50	25	0	0	25
Call 0 AM to 24 AM SD duration: median	25	0	0	25	25
Call 0 AM to 24 AM total number: median	50	0	0	0	25
Call 0 AM to 24 AM unique number: median	0	25	25	25	0
