# Supplementary material for: Identifying Objective Physiological Markers and Modifiable Behaviors for Self-Reported Stress and Mental Health Status Using Wearable Sensors and Mobile Phones: Observational Study
Source: J Med Internet Res. 2018 Jun 8;20(6):e210. doi: 10.2196/jmir.9410 (PMC6015266; doi:10.2196/jmir.9410)
Supplement: Multimedia Appendix 11 [file jmir_v20i6e210_app11.pdf]

Percentages of time each feature was selected for each fold of leave-one-cohort-out cross validation for one month MCS models.

Features used for MCS model (one month)	Leave-one-cohort-out cross validation folds				
	1	2	3	4	5
Neuroticism	50	50	50	50	50
Extraversion	0	50	100	50	0
# of naps: mean	50	50	50	0	50
Conscientiousness	0	0	50	50	50
Study duration median	50	0	50	0	50
% of positive interaction median	0	50	0	50	50
SC 0 AM to 3 AM mean amplitude: mean	0	0	67	33	33
SC sleep max amplitude: median	33	67	0	33	0
ST 0 AM to 3 AM SD: median	0	33	33	0	33
Screen 0 AM to 24 AM time stamp SD: mean	0	0	25	50	0
Screen 0 AM to 3 AM time stamp mean: mean	0	25	25	25	0
Screen 6 PM to 0 AM median duration: SD	25	25	0	0	25
Mobility 5-min distance median: median	25	25	0	25	0
